# Antifungal Pipeline

**DOI:** 10.3389/fcimb.2021.732223

**Published:** 2021-09-06

**Authors:** Todd Patrick McCarty, Peter G. Pappas

**Affiliations:** ^1^Department of Medicine, University of Alabama at Birmingham, Birmingham, AL, United States; ^2^Department of Medicine, Birmingham Veterans Affairs (VA) Medical Center, Birmingham, AL, United States

**Keywords:** antifungal, mycology, Candida, aspergillosis, fungal infections, antimicrobials

## Abstract

In many ways, fungal diseases are forgotten or neglected. Given the significantly lower frequency compared to similar bacterial etiologies across the spectrum of infectious syndromes, it makes sense that anti-bacterial agents have seen the bulk of development in recent decades. The vast majority of new antifungal medications approved for use in the past 10 years have been new versions in the same class as existing agents. Clinical mycology is crying out for new mechanisms of action in the setting of rising resistance and emergence of new organisms. Fortunately, this trend appears to be reversing. There are numerous agents in advanced stages of development offering novel dosing regimens and mechanisms of action to combat these threats. Herein we review seven antifungal agents that we hope to see come to market in the coming years to aid physicians in the treatment of mucocutaneous and invasive fungal infections.

## Introduction

Antifungal therapy has improved dramatically over the last several decades. The development of systemically available azoles such as fluconazole and itraconazole, the emergence of mold active azoles such as voriconazole, posaconazole, and isavuconazole, and finally the availability of echinocandins including anidulafungin, micafungin, and caspofungin have transformed antifungal therapy. The previous reliance on amphotericin B formulations for mist serious invasive fungal infections has shifted towards highly effective and generally less toxic agents. For example, echinocandins are now used interchangeably as initial therapy for most forms of invasive candidiasis, and the mold active azoles have virtually replaced amphotericin B as drugs of first choice for invasive aspergillosis. Despite these significant advances in therapy over the last 25 years, there remain significant gaps in the antifungal armamentarium. In particular, the emergence of antifungal resistance is a global challenge that must be addressed aggressively. The most critical examples include the global emergence of antifungal resistant *Candida glabrata* and *Candida auris* and the coemergence of azole-resistant *Aspergillus* infections. These infections have raised significant concerns about the adequacy of antifungal therapy in these situations. Moreover, there remain significant challenges among some of the rarer mold infections such as those due to *Fusarium* species and *Lomentospora* species (esp *L prolificans*) for which there are no reliably active antifungal agents.

In this review we have selected seven antifungal agents in development which at the time of this printing appear to be the furthest along in terms of development and for which there are human data from phase 1, phase 2, and/or phase 3 clinical trials. Only one of these compounds is FDA or EMA approved (FDA approved ibrexafungerp, June 2021), but several seem poised for regulatory review very soon. The agents, mechanism of action, and stage of development are summarized in **Table 1**. As a disclaimer, much of the data for some of these compounds has not been published in the public domain but is available in abstract form and/or available in the investigator brochures provided by the sponsor. Nonetheless we feel that these data are compelling enough to warrant inclusion in this detailed review of newer antifungal agents.

**Table 1:** Summary of agents.

Drug	Class	Mechanism of Action	Antifungal spectrum	Current Stage of Development	Route of Administration	Active gov identifier(s)
Rezafungin	Echinocandin	Glucan synthase inhibitor	*Candida* spp *Aspergillus* spp *P jiroveci*	Phase 3	IV only	NCT03667690 NCT04368559
Fosmanogepix	Gwt1 Inhibitor	Inhibits Gwt1 protein, disrupts GPI-anchor synthesis biopathway	*Candida* spp (except *C. krusei*) *Cryptococcus* *Aspergillus*	Phase 2	IV and PO	NCT04240886
Olorofim	Orotomide	Pyrimidine synthesis inhibitor	*Aspergillus* and less common molds	Phase 1 & 2	IV and PO	NCT04752540 NCT03583164
Ibrexfungerp	Triterpinoid	Glucan synthase inhibitor	*Candida* spp *Aspergillus* spp	Phase 3 & FDA-approved (vulvovaginal candidiasis)	PO only	NCT03059992 NCT03672292 NCT03363841
Oteseconazole	Tetrazole	14-alpha demethylase inhibitor	*Candida* spp	Phase 3	PO only	NCT03562156 NCT03561701
Encochleated Amphotericin B	Polyene	Binds to ergosterol and forms membrane pores	Broad spectrum antifungal	Phase 1 & 2	PO only	NCT04031833 NCT02629419
ATI-2307	Arylamidine	Disrupts mitochondrial membrane	Broad spectrum antifungal	Phase 1	IV only	N/A

IV, intravenous; PO, oral.

N/A, Not applicable.

## Rezafungin (Cidara)

### Background

Rezafungin is a unique echinocandin born from an iterative search to identify novel echinocandin that offered alternative dosing regimens to already approved agents (James et al., 2017). Echinocandins have become the empiric treatment of choice in invasive candidiasis while waiting for speciation and anti-fungal susceptibility testing (Pappas et al., 2016). While rezafungin does not add new utility when choosing empiric therapy, it does have the potential to expand treatment options in the setting of echinocandin resistance, noted to be increasing in frequency over time, especially with respect to *C. glabrata* (Beyda et al., 2012; Alexander et al., 2013; Pfaller et al., 2019a). The unique pharmacokinetic properties allowing for weekly, rather than daily, dosing also afford a treatment option that does not require an indwelling catheter in situations where fluconazole is either contra-indicated or ineffective.

### *In Vitro* Susceptibility Data

Robust *in vitro* data for the utility of rezafungin exists across a variety of organisms across the world. The SENTRY surveillance program evaluated the minimum inhibitory concentration of rezafungin from 2014 to 2018 for *Candida, Aspergillus*, and *Cryptococcus* (Pfaller et al., 2017a; Pfaller et al., 2017b; Pfaller et al., 2020). Results from the 2020 Pfaller study demonstrated 95-100% of *Candida* isolates were susceptible with variability based on species. Against *Aspergillus fumigatus* and *A. flavi*, rezafungin had MIC50 and MIC90 values of ≤0.008-0.015 and 0.015-0.03 respectively, equal to or within 1 dilution of the currently available echinocandins. When tested against *Cryptococcus*, the MIC50 and MIC90 were again in line with the echinocandins at >4 for both values. Multiple other groups utilizing both CLSI and EUCAST reference methods have further demonstrated this equivalence to current echinocandins (Pfaller et al., 2016; Arendrup et al., 2018b; Arendrup et al., 2019; Helleberg et al., 2020). This consistent degree of activity extends to rare species of *Candida, Saccharomyces*, and cryptic species of *Aspergillus*, including species with azole resistance (Wiederhold et al., 2018a; Toth et al., 2019). Importantly, with the rising global emergence of *Candida auris*, rezafungin retains activity against this organism, including in some instances of echinocandin resistance (Berkow and Lockhart, 2018).

### Pre-Clinical Data

The unique feature of rezafungin, in comparison to currently available echinocandins, are the stability properties that allow for dosing only once weekly instead of daily (James et al., 2017; Krishnan et al., 2017). Cellular analysis of the impact of rezafungin compared to anidulafungin with fewer potential toxicities seen with rezafungin (Ong et al., 2016). Multiple neutropenic mouse model studies have shown good predictors of efficacy against a variety of *Candida* species, including those with azole resistance, as well as activity against *Aspergillus* (Hager et al., 2018b; Lepak et al., 2018a; Lepak et al., 2018b; Lepak et al., 2019; Miesel et al., 2019). These studies confirmed the once weekly dosing schedule as well as potentially lower targets that needed to be attained compared to existing echinocandins. In addition, Monte Carlo simulations indicate a that these dosing regimens will even be able to overcome potential resistance in *fks* mutant species of *C. glabrata*, a threat of growing concern, especially in the immunocompromised patient population (Bader et al., 2018). An additional immunosuppressed mouse model showed an ability to prevent disease and increase survival when used as prophylaxis in models for *Candida, Aspergillus*, and *Pneumocystis* (Miesel et al., 2021).

### Clinical Data

Human studies have confirmed these findings. The phase 1 safety studies did not reveal any deaths or severe adverse events, and no participant withdrew from the study with participants receiving either a single dose or up to 3 weekly doses (Sandison et al., 2017). The weekly dosing regimen was confirmed with high plasma exposures. Like currently approved echinocandins, there was minimal renal excretion. This data has subsequently been used to develop a population level PK model (Lakota et al., 2018). An additional phase 1 study has shown no effect on QTc or other aspects of the cardiac electrical rhythm (Flanagan et al., 2020).

The multinational STRIVE phase 2 study was published in 2020 evaluating two different dosing strategies compared to caspofungin (Thompson et al., 2020). Two different regimens of rezafungin (400mg weekly and 400mg followed by 200mg weekly) were tested against standard dose caspofungin. The intention to treat analysis evaluated 164 patients with candidemia and 43 patients with invasive candidiasis in the absence of candidemia. Outcomes for primary and secondary endpoints were similar across the three groups and comparable to the published literature of the currently approved echinocandins.

There are currently two ongoing phase 3 studies. The ReSTORE trial is the progression of STRIVE for the treatment of candidemia and invasive candidiasis. The 400mg followed by 200mg weekly dosing regimen was chosen for final development. The ReSPECT trial is evaluating the usage of rezafungin as prophylaxis against *Pneumocystis* in addition to fungal pathogens for patients undergoing bone marrow transplantation. The comparator arm in this study is a combination of oral azole and trimethoprim-sulfamethoxazole. The same 400mg/200mg dosing regimen is being used.

## Fosmanogepix (Amplyx)

### Background

The compound currently known as fosmanogepix was first discovered by the Eisai Co. as E1210 with broad clinical activity against yeasts and molds (Nakamoto et al., 2010; Miyazaki et al., 2011). Fosmanogepix is the precursor of the active compound manogepix. The compound is a first in class anti-fungal that blocks glycosylphosphatidylinositol (GPI) production *via* inhibition of Gwt1. The GPI compounds are important for the construction of the cell wall and maintenance of homeostasis *via* a highly conserved pathway.

### *In Vitro* Susceptibility Data

Fosmanogepix has undergone susceptibility testing against a large array of yeasts and molds. Multiple studies investigating the minimum inhibitory concentrations with *Candida* show the average MIC multiple degrees lower than currently approved azoles and echinocandins. The notable exception is relatively elevated MICs to *C. krusei* while activity is retained against *C. auris* (Arendrup et al., 2018a; Hager et al., 2018a; Pfaller et al., 2019b; Arendrup et al., 2020; Arendrup and Jorgensen, 2020; Zhu et al., 2020). Good *in vitro* activity was exhibited in echinocandin non-susceptible isolate of *C. glabrata* (Pfaller et al., 2019b). Activity against *Cryptococcus neoformans* was comparable to the fluconazole (Trzoss et al., 2019). Susceptible MICs were also found with testing against *Malassezia* and *Trichosporon* (Miyazaki et al., 2011). This is a notable difference compared to echinocandins’ lack of activity against non-*Candida* yeasts.

While it is still early in the development pathway, there is some early understanding about potential for resistance. Two different potential mechanisms for resistance have been identified. One study evaluated serial passages of *C. albicans, C. parapsilosis*, and *C. glabrata* with noted development of mutations in the Gwt1 amino acid sequence resulting in significant increases in MIC over the wild type (Kapoor et al., 2019). Another study found the potential for efflux pumps in *C. albicans* and *C. parapsilosis* (Liston et al., 2020).

Multiple species of *Aspergillus* are exhibited susceptible MICs across multiple species (Miyazaki et al., 2011; Rivero-Menendez et al., 2019a; Jorgensen et al., 2020). A similar degree of activity was seen in other septated pathogenic molds in the dematiaceous mold group as well as the genera of *Fusarium, Scedosporium*, and *Lomentospora* (Rivero-Menendez et al., 2019a; Badali et al., 2021). However, when tested against the *Mucorales* family limited activity was seen (Rivero-Menendez et al., 2019a).

### Pre-Clinical Data

Fosmanogepix demonstrates *in vitro* activity against the spectrum of fungal pathogens, from yeasts to dimorphic fungi to molds leading to a wealth of animal model data. This has generated an excellent platform for clinical trials to draw on for their development.

Mouse models of both oropharyngeal and disseminated candidiasis have demonstrated good efficacy including against azole resistant strains (Zhao et al., 2018). Further investigation into a mouse model of intra-abdominal disease has shown good accumulation in infected tissues as well as superior fungal clearance compared to micafungin (Lee et al., 2021). While echinocandins have become the empiric treatment of choice for invasive candidiasis and candidemia, they have limited penetration into the central nervous system leading to concerns about treatment of azole-resistant disease when complicated by meningitis or endophthalmitis. A non-neutropenic rabbit model has shown good penetration into the eye and throughout the CNS with corresponding reductions in fungal burden of *Candida albicans* (Petraitiene et al., 2021). As rates of echinocandin resistance are rising, a mouse model of invasive candidiasis has shown retained activity of fosmanogepix against echinocandin resistant strains of *C. albicans* (Wiederhold et al., 2015). Similarly, the compound has showed comparable to better activity than anidulafungin against *C. auris* in an immunocompromised mouse model of disseminated disease (Hager et al., 2018a). Fosmanogepix and related GPI inhibitors have been shown to have equivalent activity against *Cryptococcus* spp. when compared to fluconazole in a mouse model of cryptococcal meningitis (Shaw et al., 2018). That study also showed a synergistic effect with greatly increased reductions in fungal burden in brain tissue. Further investigation into analog compounds may bring higher potency against *Cryptococcus.*

Potential utility of fosmanogepix extends beyond pathogenic yeasts to a wide variety of molds, including those without good treatment options. Multiple mouse models of pulmonary aspergillosis and disseminated fusariosis have indicated good efficacy (Hata et al., 2011; Gebremariam et al., 2019). Additional immunocompromised mouse models of pulmonary infection with *Scedosporium, Lomentospora*, and *Rhizopus* (Alkhazraji et al., 2020; Gebremariam et al., 2020). A mouse model of pulmonary *Coccidioides* showed similar decreases in fungal burden and improved survival compared to fluconazole (Viriyakosol et al., 2019).

### Clinical Data

Fosmanogepix has completed Phase 1 and Phase 2 trials in humans. The phase 1 studies examined single and multi-dose ascending regimens. Doses were well tolerated, and no significant adverse effects were observed (Hodges et al., 2017a; Hodges et al., 2017b). Bioavailability of the oral doses were >90% with minimal variation between subjects and exceeding target exposures. The phase 2 study was a single arm proof-of-concept study in candidemia (Pappas et al., 2020). The modified intention to treat analysis of 20 patients showed 80% treatment success and 15% 30-day mortality, none of which were drug related. Half of patients transitioned to oral therapy with stable pharmacokinetics. There were no treatment-related severe adverse events and no discontinuations due to adverse effects. There is an ongoing similar phase 2 study for *Aspergillus* and rare molds, and a phase 3 candidemia study is in development.

## Olorofim (F2G, LTD)

### Background

Olorofim is the first representative of a novel class of antifungals known as the orotomides. The compound was found *via* screening a library of >300,000 small molecules (Oliver et al., 2016). Upon discovery, researchers determined that the mechanism of action is through inhibition of the dihydroorotate dehydrogenase enzyme. The enzyme functions in the production of pyrimidine biosynthesis. Since discovery, it has progressed through pre-clinical studies and phase 1 human trials to currently exist in the form of an ongoing phase 2 clinical trial.

### *In Vitro* Susceptibility Data

Susceptibility testing of various molds shows very broad, but not universal anti-mold activity (Jorgensen et al., 2018). *Aspergillus* spp. nearly uniformly have low MICs to olorofim (Du Pre et al., 2018). This includes azole resistant (including *A. terreus* and cryptic species of *Aspergillus*), with generally lower MICs than currently approved anti-mold agents (Buil et al., 2017; Lackner et al., 2018; Rivero-Menendez et al., 2019b). This activity extends to *Talaromyces, Trichophyton*, and *Penicillium* (Singh et al., 2021; Zhang et al., 2021). Some dematiaceous molds like *Alternaria* and certain species of *Fusarium*. *Scedosporium* and *Lomentospora* frequently are resistant to all available treatment options, however olorofim shows potent activity with much lower MICs compared to anti-mold triazoles and amphotericin (Kirchhoff et al., 2020; Rivero-Menendez et al., 2020). The compound lacks *in vitro* activity against yeasts as well as *Mucorales* (Wiederhold, 2020).

### Pre-Clinical Data

Early effects of treatment demonstrate a fungistatic effect, however as further doses accumulate, it converts to a fungicidal effect with cell lysis observed. Mouse models show excellent survival rates against a variety of species of *Aspergillus*. A mouse model of sinus and pulmonary infection with *Aspergillus flavus* showed response and survival rates at least as good as posaconazole (Negri et al., 2018). Additionally a mouse model of CNS *Coccidioides* infection showed decreases in fungal burden that persisted longer than seen with other antifungal agents (Wiederhold et al., 2018b).

### Clinical Data

Multiple ascending single- and multiple-dose phase 1 studies have been completed (Kennedy et al., 2015; Kennedy et al., 2017a; Kennedy et al., 2017b; Kennedy et al., 2017c). Pharmacokinetics of both intravenous and oral formulations exceed the target exposure level. Olorofim was well tolerated in healthy volunteers and short infusion durations were safe while maintaining desired exposures.

A phase 2b open label study is still ongoing, recruiting patients lacking other treatment options. Multiple case reports have been reported with successful outcomes against a variety of organisms. Olorofim has successfully treated two reported cases of *Lomentospora* infections, one localized and the other disseminated, notable given the essentially pan-resistant nature of *L. prolificans* (Chen et al., 2020; Tio et al., 2020). It has also been used in combination with posaconazole for the treatment of disseminated coccidioidomycosis, previously having failed multiple different anti-fungal treatment regimens (Harvey et al., 2020).

## Ibrexafungerp (Scynexis, Formerly SCY-078)

### Background

The echinocandins are β-1,3-glucan synthase inhibitors that have come into wide use as first-line therapy for most cases of candidemia and invasive candidiasis and as salvage therapy for invasive aspergillosis. Among the three currently available echinocandins including anidulafungin, caspofungin, and micafungin, none are orally available. The lack of an oral formulation of these compounds has limited their use largely to hospitalized patient and/or those with indwelling venous access devices.

Ibrexafungerp is the first of a new class of oral glucan synthase inhibitors that are semisynthetic derivatives of enfumafungin. This is the first of the triterpinoid antifungals (Davis et al., 2020). It is structurally similar to the echinocandins, and its mechanism of action is similarly through inhibition of β-1,3-glucan synthase, but its binding site is slightly different than that of the echinocandins, but with some overlap in the binding region (Apgar et al., 2021). Through this small molecular change, ibrexafungerp maintains activity against both echinocandin susceptible and certain resistant pathogens, especially selected echinocandin resistant *Candida* spp. The compound is being developed for both oral and IV administration, but only the oral formulation has been studied in humans to date. Ibrexafungerp is well absorbed orally and is generally considered to be fungicidal. The oral formulation was recently approved by FDA for treatment of vulvovaginal candidiasis (VVC).

### *In Vitro* Susceptibility Data

*In Vitro* studies demonstrate significant activity against most common *Candida* species including several resistant strains such as *Candida glabrata* and *Candida auris* (Schell et al., 2017a; Ghannoum et al., 2020). Moreover, it demonstrates excellent *in vitro* activity *versus* both wild-type and azole-resistant strains of *Aspergillus* (Lamoth and Alexander, 2015). It is also active against *Pneumocystis jiroveci* but this has only been demonstrated in animal models and not in human trials. The compound does not show significant activity *versus* other important fungal pathogens such as *Fusarium* for the *Mucorales*. It does retain activity against *Candida* FKS-1 and FKS-2 mutations including those demonstrated in certain strains of *Candida glabrata* (Nunnally et al., 2019).

### Clinical Data

There are several completed and ongoing phase 2 and phase 3 clinical trials with ibrexafungerp. In an unpublished study of vulvovaginal candidiasis, among 96 subjects oral ibrexafungerp was superior to fluconazole at end of therapy, and at the end of 4-month follow-up with an overall lower recurrence rate (4% *vs* 15%). In the first published study of a multicenter clinical trial for the treatment of invasive candidiasis, oral step-down therapy with ibrexafungerp was compared to oral fluconazole after an induction course of a parenteral echinocandin for 3 to 10 days. The study achieved its main objective in providing target exposure to ibrexafungerp in over 80% of subjects; the compound also demonstrated excellent efficacy with no dose-limiting adverse events (Spec et al., 2019). Adverse events which were observed were limited to gastrointestinal distress including nausea, vomiting, abdominal bloating, and diarrhea. In the second multicenter trial, women with acute VVC were randomized to receive ibrexafungerp 300 mg bid or fluconazole 150 mg single dose (DOVE Trial). Overall, 51 subjects received ibrexafungerp and 24 received fluconazole. At day 10, the clinical cure rate for ibrexafungerp and fluconazole were 51.9% and 58.3%, respectively; at day 25, patients with no signs or symptoms were 70.4% and 50.0% among ibrexafungerp and fluconazole recipients respectively (Angulo et al., 2019). During the study ibrexafungerp patients required less antifungal rescue medications compared with fluconazole (3.7% *vs* 29.2%, respectively). Ibrexafungerp was well-tolerated, with the most common treatment-related adverse events being mild gastrointestinal events. A recently completed phase 3 trial comparing ibrexafungerp 300 mg orally twice daily for one day *versus* placebo among 286 women with acute VVC (VANISH 303) demonstrated superiority of ibrexafungerp *versus* placebo (50% *vs* 29%) (Schwebke J submitted CID). Finally, there is an ongoing phase 2 combination study of ibrexafungerp plus voriconazole *versus* voriconazole monotherapy for treatment of invasive pulmonary aspergillosis (SCYNERGIA Study).

There are two ongoing phase 3 trials of ibrexafungerp. The largest enrolling study to date is the FURI study, which evaluates efficacy and safety of ibrexafungerp among patients with severe fungal infection and who are refractory to or intolerant of standard antifungal agents. Over 100 patients have been enrolled into this open label trial, but no data have been published as the study is ongoing. The CARES Study is an open label trial in the United States and India evaluating the use of ibexafungerp in 30 subjects with *Candida auris* infection; this study is nearing completion of enrollment. A large randomized and double-blind phase 3 trial is being planned which will compare ibrexafungerp and fluconazole as oral stepdown therapy for subjects receiving no more than 3 days of echinocandin induction therapy for candidemia and/or invasive candidiasis.

## Oteseconazole (Mycovia, VT-1161)

### Background

Oteseconazole (VT-1161) is the first of several tetrazole agents which have in common a much greater affinity for fungal CYP51 compared to human cytochrome enzymes. The compound is designed with the goal of greater selectivity, fewer side effects, and improved efficacy compared to currently available azoles. Some studies have suggested that the affinity for fungal CYP51 exceeds 2000 fold selectivity over the human enzyme counterpart (Hoekstra et al., 2014; Warrilow et al., 2014). Based on this observation that one would expect fewer drug-drug interactions with this class of compounds but also less direct toxicity. The target enzyme of the tetrazoles (14-alpha demethylase), including oteseconazole is the same as for other azoles, however the affinity for the target enzyme is greater than for the triazoles such as fluconazole.

### *In Vitro* Susceptibility Data

This tetrazole demonstrates broad activity against most *Candida* species, dermatophytes, certain endemic fungi such as *Coccidioides* species, and selected *Mucorales* species. *In Vitro* studies suggest that the compound is active against fluconazole-resistant *Candida* strains such as *Candida krusei* and *Candida glabrata*, demonstrating activity similar to other triazoles such as voriconazole and posaconazole (Schell et al., 2017b; Nishimoto et al., 2019a; Nishimoto et al., 2019b).

### Clinical Data

In clinical trials performed to date, this orally administered oteseconazole has been examined among women for both acute and recurrent vulvovaginal candidiasis (VVC) and among subjects with onychomycosis. In a randomized placebo-controlled, double-blind, dose-ranging study of women with recurrent VVC, women receiving any of 4 regimens of VT 1161 experienced significant reduction in recurrent VVC compared to placebo (Brand et al., 2018). The compound was very well tolerated, leading to a few early discontinuations during the study when administered on a weekly basis at doses ranging from 150 to 300 mg weekly. In a second phase 2 trial involving women with acute VVC, patients were randomized to receive 3 different regimens of VT-1161 compared to standard of care with a single dose fluconazole 150 mg (Brand et al., 2020). Approximately 80% of women receiving VT- 1161 experienced therapeutic cure, compared to 62.5% in the fluconazole group (differences were not statistically significant). At 3 and 6 months following completion of therapy, no participants in the VT-1161 groups *versus* 28.5 and 46.1% in the fluconazole groups, respectively, developed mycologically confirmed recurrence of vaginitis. There were no serious adverse events leading to discontinuation of study medication. To date, over 850 women have participated in clinical trials involving oteseconazole for VVC. There is a single phase 2 randomized double-blind, placebo-controlled study of VT 1161 for treatment of onychomycosis (Elewski et al., 2021). This study compared 1 of 4 dosing regimens of oral VT 1161 to placebo, each administered for 10 to 22 weeks. The active regimens included 300 mg or 600 mg VT- 1161 administered daily for 2 weeks, followed by weekly dosing with the randomized dose for either 10 or 22 weeks. A placebo arm was included. Cure rates in the placebo arm were 0%, compared to 32 to 42% in the VT 1161 treatment arms. Study drug was well-tolerated in this trial involving 259 patients.

As an agent that is only available in an oral formulation, it remains unclear whether oteseconazole will have a role in the treatment of more serious invasive fungal infections such as candidemia and invasive candidiasis. At present there are no studies underway to examine this compound in these clinical settings. Nonetheless, the potential for a systemically administered tetrazole compound with significantly less potential for drug-drug interactions and direct toxicity is very appealing, and it could represent a significant step forward for the azole class of drugs. Oteseconazole is currently under FDA consideration for approval as an agent for the treatment of recurrent VVC.

## Encochleated Amphotericin B (Matinas, MAT2203)

### Background

Intravenously administered amphotericin B deoxycholate (AmB-D) is a traditional pillar of therapy for serious fungal infections due to its broad antifungal bacterial activity and very low rates of antifungal resistance. However, intravenously administered a AmB-D has significant toxicities including infusion related reactions, nephrotoxicity, hyperkalemia, hypomagnesemia, and anemia. Lipid formulations of AmB are a significant improvement over AmB-D in terms of drug-associated renal toxicity, but there remain significant electrolyte disturbances with their use, such as hypokalemia and hypomagnesemia. Additionally, intravascular volume expansion with normal saline is recommended with their use to minimize renal toxicity. Finally, infusion-related toxicity such as fever, chills and hypotension are seen with lipid formulations of AmB. In order to avoid these significant toxicities, an orally administered encochleated AmB-d (C-AmB) has been developed as a novel lipid nanocrystal which is designed for oral therapy for serious fungal infections. C-AmB is comprised of a solid lipid bilayer along with calcium which are rolled into a spiral form where amphotericin B molecules (or other biologically active molecules) are entrapped (Segarra et al., 2002). The drug is made biologically available when the cochlea are taken up by a phagocytic cell and the gradient between the high calcium concentration in the cochlea and the lower cytoplasmic calcium concentration triggers the cochlea to unfold and open thereby releasing amphotericin B molecules. Moreover, the cochlea structure provides protection against degradation in unfavorable environments such as low the pH in the stomach, while allowing for targeted intracellular delivery into macrophages and reticuloendothelial cells.

### *In Vitro* Activity

The *in vitro* activity of AmB-d is well documented and need not be recounted here. The encochleated formulation of AmB-d demonstrates retention of the broad-spectrum activity of the parent compound in murine models of candidiasis and aspergillosis (Zarif et al., 2000; Delmas et al., 2002).

### Preclinical Development

C-AmB has been studied in murine models of candidiasis aspergillosis and cryptococcosis (Zarif et al., 2000; Delmas et al., 2002; Lu et al., 2019). Animal studies have suggested good tolerability and effective treatment of murine models of these infections with demonstration of therapeutic levels of AmB the brain tissue, liver and spleen (Segarra et al., 2002).

### Clinical Development

To date, human studies have been limited several phase1 and 2 trials of safety, tolerability and efficacy. A recently published phase 1 study among 35 volunteers with a history of recovered HIV-associated CNS cryptococcosis (EnACT 1) demonstrated that the drug is generally tolerated well up to a dose of 1.5 g daily, above which significant bloating abdominal pain and diarrhea were common adverse effects (Skipper et al., 2020). It is notable that among all the human studies performed to date with C–AmB, the traditional side effects of parenteral amphotericin B including hyperkalemia, anemia, renal failure administration associated side effects have not been seen.

Three phase 2 studies have been performed or are underway among adults with a variety of conditions. Four adults with hypereosinophilia and IL-17 dysfunction who have a history of chronic oropharyngeal candidiasis and esophageal candidiasis due to azole-resistant organisms and refractory to other forms of therapy have been enrolled into one trial. Three of these for subjects have had dramatic clinical improvement following variable lengths of therapy lasting up to 48 months. Complete suppression of recurrence was noted in all three of these patients (personal communication MS Lionakis). A phase 2 trial comparing C-AmB (orally) to conventional AmB (intravenously) as induction therapy among patients with newly diagnosed HIV-associated cryptococcal meningitis is currently enrolling subjects (EnACT 2). In this trial C-AmB is being administered as primary/induction therapy and compared to AmB-d intravenously (CT.gov NCT04031833). Measures of outcome include clinical and mycologic (cerebrospinal early fungical activity). Results of this ongoing are unavailable at this time.

The promise of C-AmB is the potential for broad-spectrum antifungal activity delivered by the oral route to the target organ with very little, if any, of the typical toxicity associated with intravenously administered AmB regardless of formulation (lipid *versus* deoxycholate). Larger comparative clinical trials will be necessary to determine if this agent has a potential role in serious systemic fungal infections, but data at this point show significant promise in selected populations.

## ATI-2307 (Appili Therapeutics)

### Background

The novel anti-fungal agent ATI-2307 is an arylamidine compound that has a new mechanism of action among existing antifungal agents. The agent inhibits mitochondrial function and causes collapse of membrane potential in fungal cells preferentially (Shibata et al., 2012). The compound selectively disrupts mitochondrial function through inhibition of respiratory chain complexes (Yamashita et al., 2019). ATI-2307 enters the fungal cell *via* active transport and selectively inhibits the fungal mitochondrial membrane potential with >1000x selectivity compared to rat hepatocytes, leading to a decrease in the production of ATP and growth inhibition *via* energy depletion.

There is little metabolic degradation of the drug; it remains in tissues, generally bound to mitochondrial proteins, for days to weeks. Serum half-life is measured in hours, and dosing to achieve optimal serum levels in humans is probably optimized with dosing intravenously every twelve hours. ATI-2307 is excreted slowly in the urine and feces. It does not accumulate in serum with twice daily dosing up to at least up to 20 mg twice daily for 21 days (Appili Therapeutics, LTD). This compound has little potential for cytochrome P450 interaction, thus drug-drug interaction potential is low. At present, ATI-2307 is only available as a parenteral (intravenous) formulation.

### *In Vitro* Susceptibility Data

Pre-clinical *in vitro* studies have shown a high degree of activity against a variety of pathogenic fungi: all major *Candida* species (including *C. auris*, and other fluconazole and echinocandin-resistant *Candida* spp), *Cryptococcus*, *Aspergillus*, and *Fusarium*. Antifungal activity is similar to or higher than currently approved agents (Mitsuyama et al., 2008; Fujifilm Toyama Chemical Company LTD, 2019; Sakagami et al., 2019). Additionally, it retains activity in the setting of antifungal drug resistance in these organisms. *In vivo* studies in murine models for systemic candidiasis across multiple species of *Candida* (including drug resistance) demonstrates a lower minimum effective dose of ATI-2307 compared to fluconazole, micafungin, and amphotericin B (Wiederhold et al., 2016; Wiederhold et al., 2020).

### Clinical Development

To date, ATI-2307 has not been administered to patients with proven or probable *Candida* infection. ATI-2307 has tremendous potential given its broad activity against all tested *Candida* species including *C. auris* and other AFR *Candida* species. Moreover, the reported MIC’s against these susceptible strains are sufficiently low (generally < 0.01 mcg/ml) that effective serum levels of ATI-2307 should be readily achieved with every 12 to 24-hour dosing as proposed.

Currently developed as an intravenous formulation, three Phase I studies have been completed in healthy adults (these data are available through Appili Therapeutics). A single ascending dose study (ranging from 0.125mg to 5mg) compared ATI-2307 to placebo in 55 subjects demonstrated no serious adverse events (SAEs) related to the study drug and similar numbers of treatment-emergent adverse events (TEAEs) compared to placebo. A second phase 1 study involving single (10 or 20mg) or multiple (2.5, 5, or 10mg daily for 21 days) doses compared to placebo in 48 subjects and again showed no SAEs related to study drug. A final Phase I multiple dose study involving 27 subjects examined higher doses (10, 15, or 20mg/day) of ATI-2307 administered twice daily for 21 days and demonstrated no drug-associated SAEs. Three subjects were withdrawn from the study due to TEAEs, not all of which were thought to be related to the study drug. The most common types of treatment related adverse events were neurosensory events, very modest tachycardia for approximately 20-30 minutes during the infusion, headache, and dysgeusia (Fujifilm Toyama Chemical Company LTD, 2019). All adverse events (AEs) were transient and did not require treatment. No AEs were noted at the 3-month or 6-month follow-up for the respective studies which cumulatively enrolled 130 subjects. Pharmacokinetic data from these studies show 50-55% protein binding in the serum. There were no noted inhibitory effects on cytochrome pathway, with minimal concentrations of metabolites detected in the plasma, urine, and feces. None of these human data are not published in the public domain but data are available in the investigator’s brochure provided by the drug manufacturer. This compound has not been administered to any participants under the age of 18. Similarly, limited drug-drug interaction data are available based on existing Phase I studies, but as ATI-2307 is not metabolized through the cytochrome pathway, we expect few drug-drug interactions.

The clinical role for ATI–2307 is unclear, however its broad *in vitro* activity against a host of important fungal pathogens, including multidrug resistant organisms, could translate into an critical role for this compound, especially for fungal infections due to drug-resistant organisms such as *Candida auris*, multidrug-resistant *Candida glabrata*, and azole-resistant *Aspergillus* species. A phase 2 study comparing ascending doses of ATI–2307 to standard of care with an echinocandin among patients with candidemia due to *Candida auris* and other antifungal resistant *Candida* species is being planned.

## Summary

We believe that the future antifungal therapy is very bright. The development of multiple agents, many of which demonstrate novel mechanisms of action and which demonstrate significant activity *versus* a very a broad array of fungal pathogens, including those known to be resistant to conventional agents, is quite promising. It should not be surprising that each of these compounds has been developed initially by a smaller biotech firm that has been incentivized through governmental programs such as the GAIN (Growing American Innovation Now) Act (www.epw.senate.gov/public/index.cfm/2019/10/senators-introduce-growing-american innovation now) and similar programs which provide prolonged patent protection, fast-track status for regulatory approval for compounds meeting certain criteria, and the ability to develop and market a compound for very uncommon infections. In our opinion, this type of incentivized approach to new antifungal development is essential given the relatively small frequency of human cases due to these pathogens, and critically important towards maintaining a therapeutic edge on existing and emerging fungal pathogens globally.

## Author Contributions

TM and PP jointly developed the outline and wrote the manuscript. Both authors contributed to the article and approved the submitted version.

## Conflict of Interest

TM - research grant support from Amplyx, Cidara; Consulting - T2Biosystems PP - Research support: Merck, Amplyx, Scynexis, Gilead, Astellas, Mayne, Cidara; Consulting, DRC: Matinas, Cidara, Mayne, Scynexis, Appili; Speakers bureau: None; Equities: None.

The remaining author declares that the research was conducted in the absence of any commercial or financial relationships that could be construed as a potential conflict of interest.

The handling editor declared a shared consortium with one of the authors PP at time of review.

## Publisher’s Note

All claims expressed in this article are solely those of the authors and do not necessarily represent those of their affiliated organizations, or those of the publisher, the editors and the reviewers. Any product that may be evaluated in this article, or claim that may be made by its manufacturer, is not guaranteed or endorsed by the publisher.
